# *Bacteroides thetaiotaomicron* and *Lactobacillus johnsonii* modulate intestinal inflammation and eliminate fungi via enzymatic hydrolysis of the fungal cell wall

**DOI:** 10.1038/s41598-020-68214-9

**Published:** 2020-07-13

**Authors:** Rogatien Charlet, Clovis Bortolus, Boualem Sendid, Samir Jawhara

**Affiliations:** 1 CNRS, UMR 8576 - UGSF - Unité de Glycobiologie Structurale Et Fonctionnelle, INSERM U1285, 59000 Lille, France; 2 University of Lille, 1 place Verdun, 59000 Lille, France; 3 CHU Lille, Service de Parasitologie Mycologie, Pôle de Biologie Pathologie Génétique, 59000 Lille, France

**Keywords:** Fungal host response, Dysbiosis, Gastrointestinal models

## Abstract

Alterations to the gut microbiota can cause an amplification of the inflammatory response to intestinal pathogens. We assessed the effect of *Bacteroides thetaiotaomicron* and *Lactobacillus johnsonii* on the elimination of *Candida* species and whether restoration of these two anaerobic bacteria could attenuate the development of colitis in mice. In this study, *L. johnsonii* and *B. thetaiotaomicron* interacted directly with *Candida* species and induced a degradation of the fungal cell wall, mediated via chitinase-like and mannosidase-like activities, which promoted the inhibition of *Candida* species growth. In the DSS-induced colitis model, oral administration of *L. johnsonii* and *B. thetaiotaomicron* to mice reduced the overgrowth of *Escherichia coli*, *Enterococcus faecalis* and *Candida glabrata* populations and resulted in a significant reduction in inflammatory parameters. *L. johnsonii* and *B. thetaiotaomicron* decreased pro-inflammatory mediators and enhanced the anti-inflammatory cytokine response with high TLR9 expression and chitinase-like protein-1 activation, which promoted the elimination of *C. glabrata* from the gut. Overall, these findings provide evidence that *L. johnsonii* and *B. thetaiotaomicron* decrease the development of colitis mediated by TLR9 and promote the elimination of *C. glabrata* from the gut via chitinase-like and mannosidase-like activities.

## Introduction

The gut microbiota plays a crucial role in protecting the host from colonization/infection by pathogens and in stimulating/modulating the immune system^1,2^. Alterations to the gut microbiota can occur via changes in the diversity of the bacterial community or by microbiota-host interactions and can be directly correlated with several diseases, in particular inflammatory bowel disease (IBD), which includes Crohn’s disease (CD) and ulcerative colitis (UC)^3^. Ulceration from CD can occur anywhere in the gastrointestinal tract while the hallmark of UC is ulceration beginning in the rectum and limited to the colon^4^. Although the pathogenesis of IBD is unknown, different studies have reported that changes in gut microbiota biodiversity, genetic susceptibility, environmental factors and the immune system are all involved^5–7^. Numerous studies have shown that the quality and composition of the microbiota are altered in IBD patients, with a reduction of Firmicutes and Bacteroidetes and an increase in Proteobacteria and Enterobacteria^8^. These alterations to the gut microbiota can cause an amplification of the inflammatory response to intestinal pathogens and trigger a range of mechanisms including increased epithelial permeability and decreased luminal IgA concentrations^9^. Additionally, ineffective bacterial clearance leads to excessive Toll-like receptor (TLR) stimulation, secretion of pro-inflammatory cytokines and activation of the immune response. In a model of dextran sulphate sodium (DSS)-induced colitis, overgrowth of *Escherichia coli* and *Enterococcus faecalis* populations, which are representative of the Enterobacteria and Proteobacteria phyla respectively, are associated with high levels of expression of TLR and pro-inflammatory cytokines^10^. Furthermore, increased *E. coli* or *E. faecalis* populations are also associated with clinically active CD^11–13^. In contrast, *Bacteroides thetaiotaomicron* and *Lactobacillus johnsonii,* which are representative of Bacteroidetes and Firmicutes respectively, are reduced during the devolvement of colitis and this reduction is more pronounced for *L. johnsonii* in colitic mice challenged with the opportunistic yeast *Candida glabrata*^14,15^.

Like *Candida albicans*, *C. glabrata* is an endosaprophytic yeast that can cause invasive fungal infection (IFI)^16^. Systemic *C. glabrata* infections are associated with higher mortality than *C. albicans* infections^16,17^. *C. glabrata* interacts with the host at the level of the fungal cell wall, which consists mainly of polysaccharides associated with proteins and lipids^18^. Fungal polysaccharides are released into the blood during infection and their detection enables the early diagnosis of IFI^19^. Clinical and experimental studies have shown that infection by *Candida* species can generate anti-glycan antibodies known as ASCA (anti-*Saccharomyces cerevisiae* mannan antibodies), ACCA (anti-chitobioside carbohydrate antibodies) and ALCA (anti-laminaribioside carbohydrate antibodies), which are described as serological markers of CD, suggesting a link between CD gut dysbiosis and endogenous opportunistic yeast species^20,21^. The DSS-induced colitis model promoted an abundance of *C. glabrata* which in turn increased the severity of inflammation leading to intense bleeding and diarrhoea, colonic epithelial damage and inflammatory cell infiltrates^18^.

Given that *B. thetaiotaomicron* and *L. johnsonii* populations are highly affected during the development of colitis and overgrowth of *C. glabrata*, we assessed whether restoration of these two anaerobic bacteria can attenuate the development of colitis and *C. glabrata* growth^14,15^. We then measured inflammatory parameters, changes in aerobic bacteria populations and modulation of receptor and cytokine expression. In addition to the DSS-colitis model, the direct effect of these two bacteria on *C. glabrata* growth/viability were analysed in vitro.

Our results show that *L. johnsonii* and *B. thetaiotaomicron* interact directly with *Candida* species and induce degradation of the fungal cell wall, mediated via chitinase-like and mannosidase-like activities, which promotes the inhibition of *Candida* species growth. In the DSS-induced colitis model, oral administration of *L. johnsonii* and *B. thetaiotaomicron* to mice restored the imbalance between aerobic and anaerobic populations and resulted in a significant reduction in inflammatory parameters, revealed by a decrease in pro-inflammatory mediators, and enhanced the anti-inflammatory cytokine response with high TLR9 expression and chitinase-like protein-1 activation, which promoted *C. glabrata* elimination from the gut.

## Results

### Impact of *B. thetaiotaomicron* and *L. johnsonii* on *C. glabrata* and *C. albicans* growth

**Figure 1:**
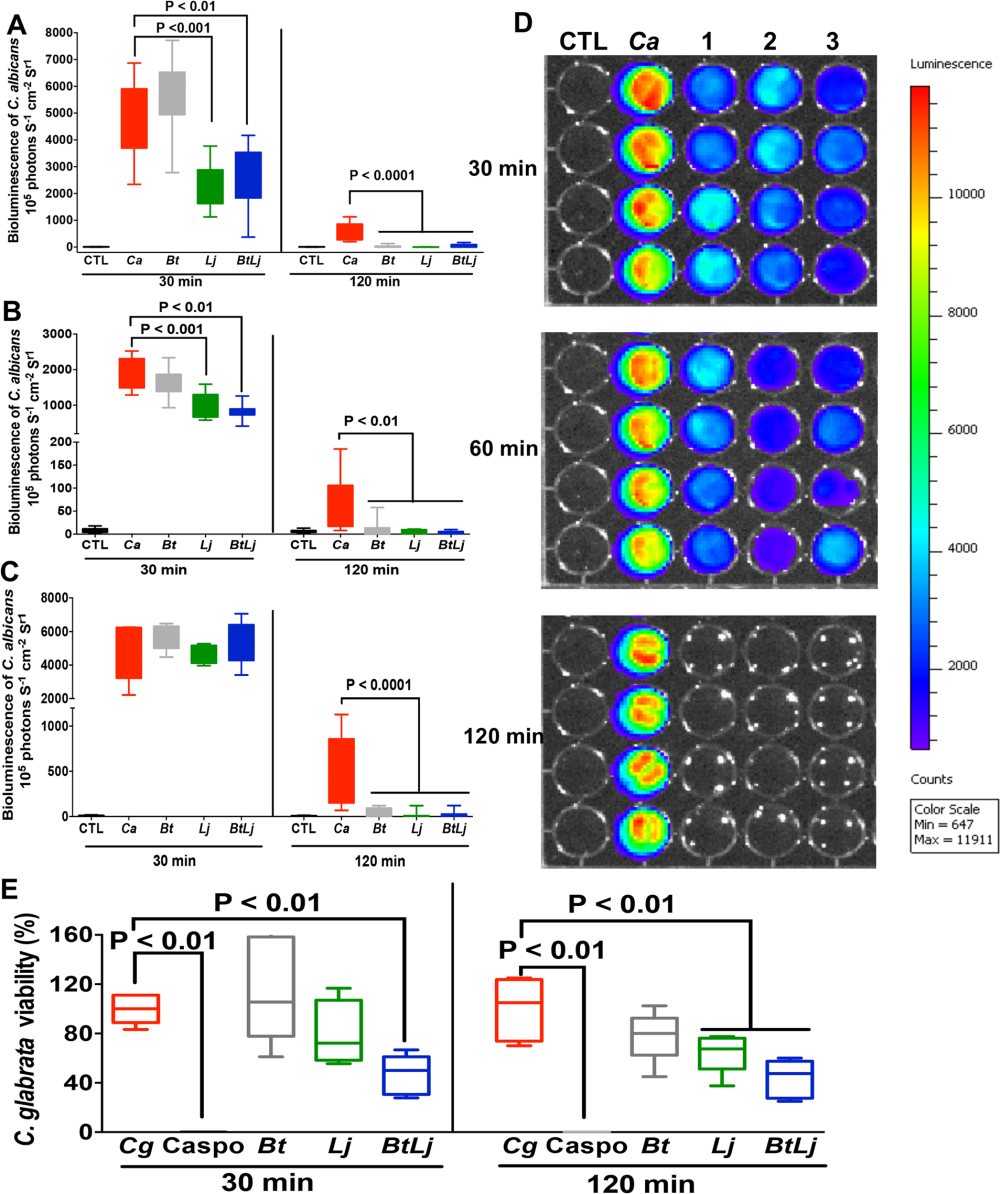
Antifungal effect of *B. thetaiotaomicron* and *L. johnsonii* on *C. albicans* and *C. glabrata*. (**A–C**) A bioluminescent *C. albicans* strain was treated with either *B. thetaiotaomicron* and/or *L. johnsonii* at a yeast-bacteria ratio of 1:1, 5:1 and 1:5, respectively, and monitored at 30 and 120 min. CTL corresponds to PBS and substrate only. Ca corresponds to *C. albicans* alone without bacteria. CaBt, CaLj and CaBtLj correspond to co-incubation of *C. albicans* with *B. thetaiotaomicron* and/or *L. johnsonii*. The results were obtained from three independent experiments. (**D**) Visualization of the bioluminescence of *C. albicans* in real-time. Bioluminescence of *C. albicans* incubated with *B. thetaiotaomicron* and/or *L. johnsonii* at a ratio of 1:1 and monitored at 30, 60 and 120 min. Line CTL: PBS without yeasts. Line Ca: *C. albicans* alone. Line 1 corresponds to *C. albicans* treated with *L. johnsonii* at a ratio of 1:1. Line 2 represents *C. albicans* treated with both *B. thetaiotaomicron* and *L. johnsonii* at a bacteria-yeast ratio of 1:1. (**E**) Viability of *C. glabrata* in the presence of *B. thetaiotaomicron* and/or *L. johnsonii*. *C. glabrata* was challenged with *B. thetaiotaomicron* and/or *L. johnsonii* at a ratio of 1:1. The results were obtained from three independent experiments.

**Figure 2:**
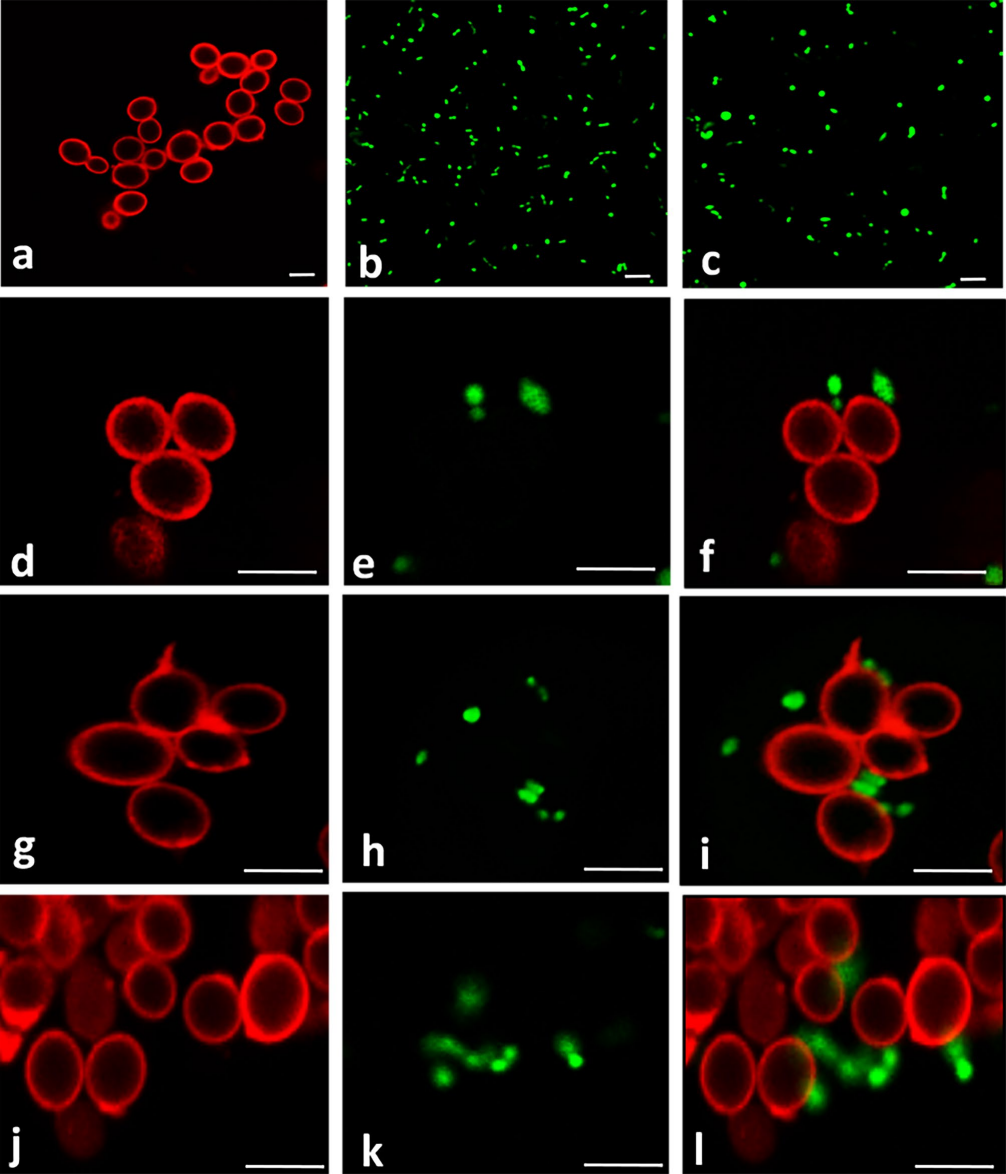
Confocal microscopy of the interaction between *C. glabrata*, *B. thetaiotaomicron* and *L. johnsonii*. Controls (**a**) *C. glabrata* alone labelled with Con A, (**b**) *B. thetaiotaomicron* alone labelled with calcein, (**c**) *L. johnsonii* alone labelled with calcein. (**d**) Co-incubation of *C. glabrata* with (**e**) *B. thetaiotaomicron*: (**f**) merged images. (**g**) Co-incubation of *C. glabrata* with (**h**) *L. johnsonii*: (**i**) merged images. (**j**) Co-incubation of *C. glabrata* with (**k**) both *B. thetaiotaomicron* and *L. johnsonii*: (**l**) merged images. The scale bars correspond to 5 μm.

Based on our previous observations that intestinal inflammation and *Candida* species promoted a significant decrease in anaerobic bacteria in a murine model of DSS-induced colitis, in particular *B. thetaiotaomicron* and *L. johnsonii*, we first assessed whether *B. thetaiotaomicron* + *L. johnsonii* could inhibit the growth of *C. albicans* and *C. glabrata*, which are the two most frequent causes of human yeast infections^14,15^. To determine the viability of *C. albicans* in real-time, a bioluminescent *C. albicans* strain was incubated with either *B. thetaiotaomicron* or *L. johnsonii* or both for different periods of time and at different concentrations. We observed a significant decrease in bioluminescence of *C. albicans* in the presence of *L. johnsonii* or both bacterial strains starting a few minutes after co-incubation of the bacteria with yeasts (at a ratio of 1:1 or 1:5). The immediate effect of these bacteria on *C. albicans* was not observed when the ratio was 5:1. Furthermore, *C. albicans* bioluminescence decreased significantly after 2 h co-incubation with the two bacterial strains at all ratios when compared to *C. albicans* unchallenged with bacteria (Fig. 1A–D). The effect of these bacteria on *C. glabrata* growth was assessed using culture methods based on agar plates. A significant decrease in *C. glabrata* colonies was observed in the presence of these bacteria after 2 h co-incubation (Fig. 1E). Confocal microscopy revealed that *B. thetaiotaomicron* and *L. johnsonii* interacted closely with the *C. glabrata* cell wall (Fig. 2).

### Antifungal activity of bacterial enzymes against *C. glabrata*

**Figure 3:**
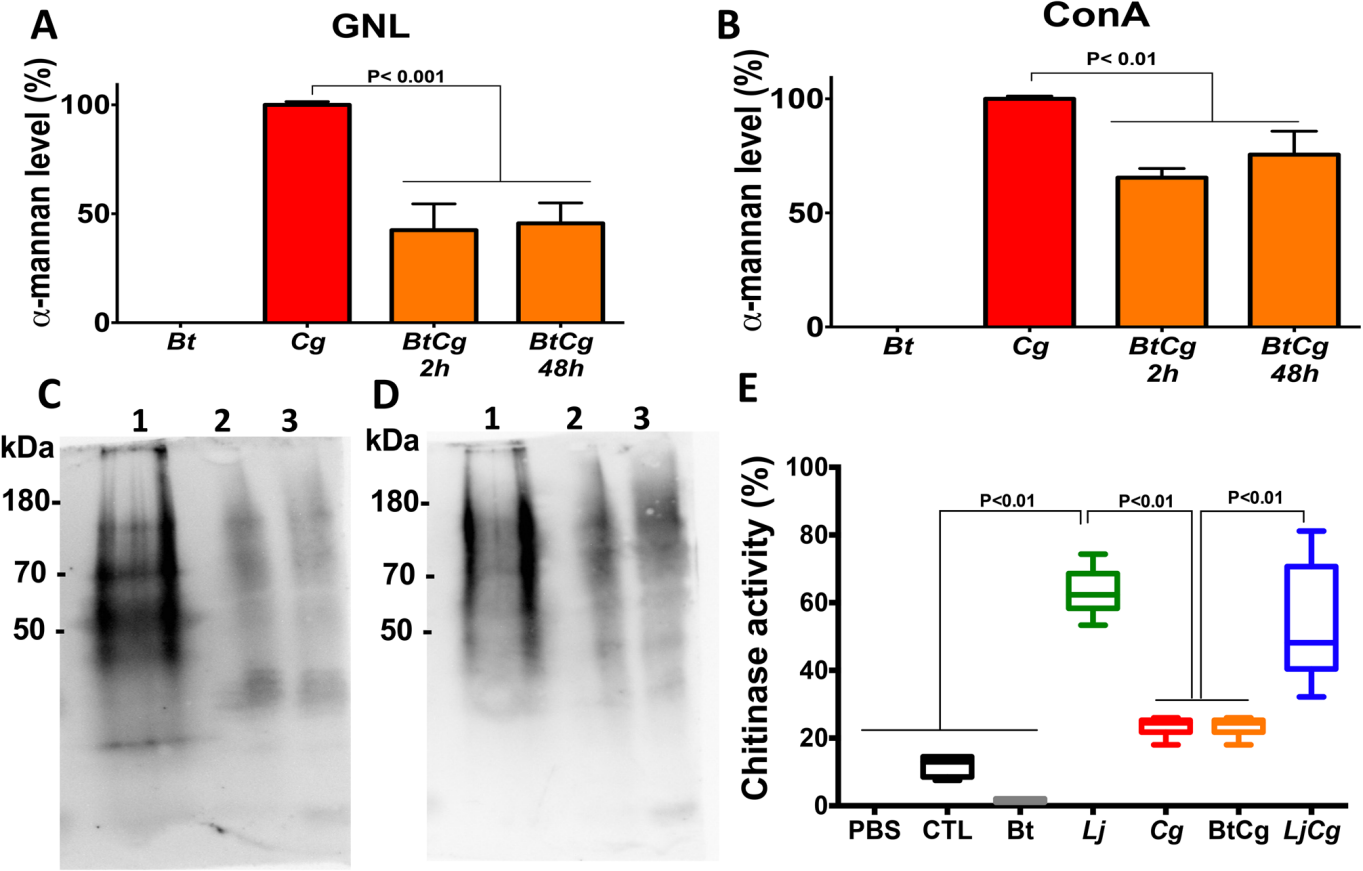
Antifungal activities of *B. thetaiotaomicron* and *L. johnsonii* against *C. glabrata.* (**A** and **B**) Determination of *C. glabrata* cell wall α-mannan levels after co-incubation of yeasts + bacteria at a ratio of 1:1. (**C** and **D**) Western blot analysis of *C. glabrata* cell wall α-mannan labelled with either GNL or ConA, respectively. Line 1: *B. thetaiotaomicron* extract*.* Line 2: *C. glabrata* extract. Line 3: extract of both *C. glabrata* and *B. thetaiotaomicron* incubated for 2 h. Line 4: extract of both *C. glabrata* and *B. thetaiotaomicron* incubated for 48 h. Different exposures of the blots were performed (Supplementary data). (**F**) Determination of chitinase activity of *L. johnsonii* and *B. thetaiotaomicron* in the presence or absence of *C. glabrata*.

After monitoring in real-time how *B. thetaiotaomicron* and *L. johnsonii* were able to interact directly with *Candida* and inhibit fungal growth, we then assessed whether *B. thetaiotaomicron* and *L. johnsonii* were able to induce cell wall degradation of *C. glabrata*, α-mannan and chitin (Fig. 3). In contrast to *L. johnsonii* which did not show any mannosidase-like activity against *C. glabrata, B. thetaiotaomicron* was able to induce degradation of *C. glabrata* α-mannan in the cell wall after 2 h co-incubation (Fig. 3A–D). To confirm that mannans are degraded by *B. thetaiotaomicron, C. glabrata* mannan was extracted and co-incubated with *B. thetaiotaomicron*. Mannans derived from *C. glabrata* alone form a smear in SDS-acrylamide gels as do all other heavily glycosylated yeast glycoproteins^22,23^ (Supplementary data). This mannan smear was no longer visible after incubation of *B. thetaiotaomicron* with mannans. These data correlate closely with those showing degradation of cell wall mannan after co-incubation of *C. glabrata* cells with *B. thetaiotaomicron* and support the idea that *B. thetaiotaomicron* is able to induce degradation of *C. glabrata* cell wall mannan and exert a mannosidase-like activity that promotes *C. glabrata* elimination. In terms of chitin, *L. johnsonii* exhibited chitinase-like activity, which was correlated with the elimination of *C. glabrata.* In contrast, *B. thetaiotaomicron* did not show any chitinase-like activity against *C. glabrata* (Fig. 3F).

### Impact of *B. thetaiotaomicron* and *L. johnsonii* on intestinal inflammation, *C. glabrata* elimination and modulation of bacteria populations

In order to assess whether these two bacteria exert antifungal and/or inflammatory properties, the effect of *B. thetaiotaomicron* and *L. johnsonii* on intestinal inflammation and *C. glabrata* elimination was assessed in the DSS-induced colitis model. Mice were administered a single oral dose of *C. glabrata* and were exposed to DSS treatment for 2 weeks in order to induce acute colitis. These two bacteria were then administered orally to mice for 5 days after *C. glabrata* challenge.

**Figure 4:**
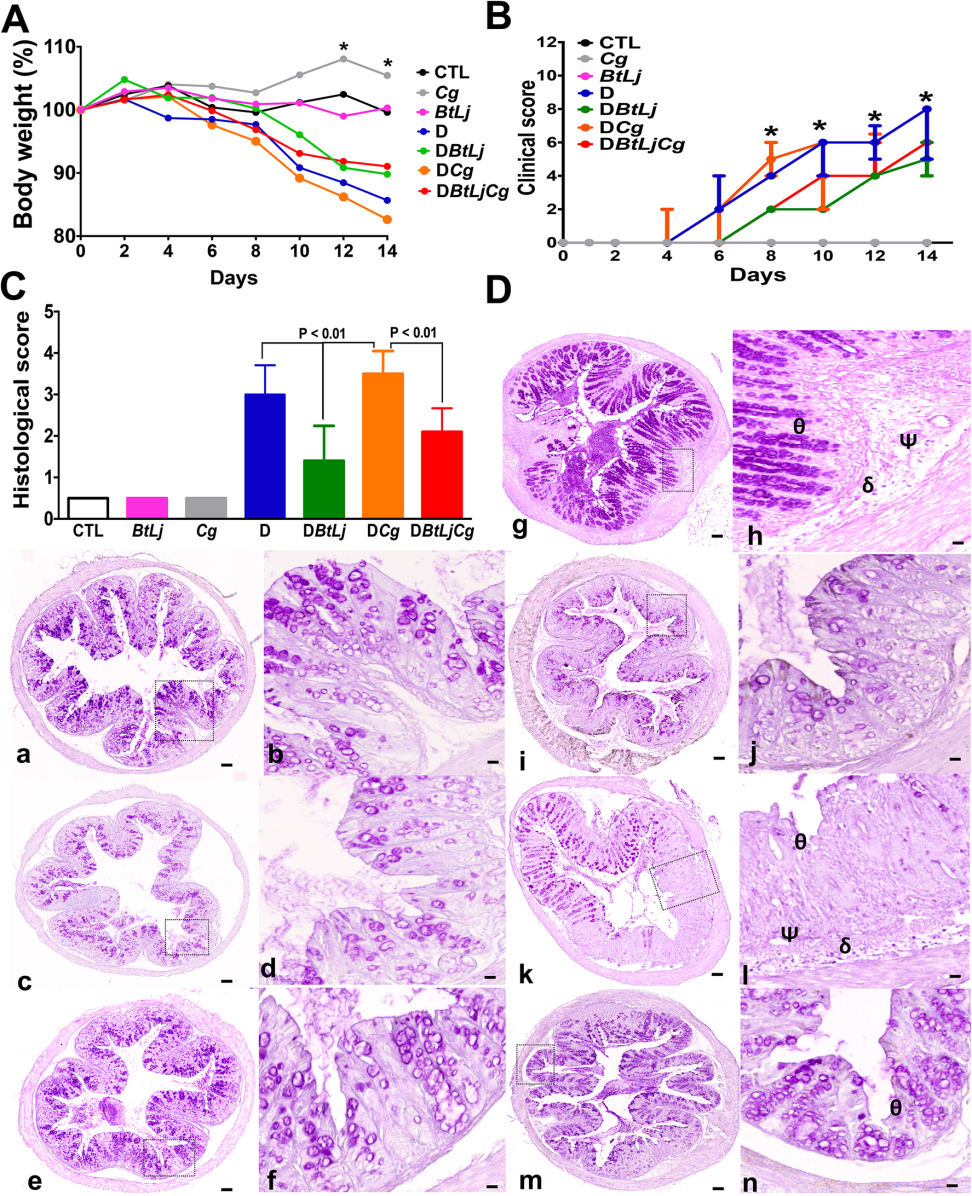
Determination of inflammatory parameters in the DSS-induced colitis model. (**A**) Body weight. Control groups correspond to CTL (water), Cg (*C. glabrata*) and BtLj (*B. thetaiotaomicron* + *L. johnsonii*). Experimental groups correspond to D (DSS), DBtLj (DSS + *B. thetaiotaomicron* + *L. johnsonii*) and DBtLjCg (DSS + *B. thetaiotaomicron* + *L. johnsonii* + *C. glabrata*). Data are the mean ± SD of eight mice per group from two independent experiments. * p < 0.05, D and DCg groups *vs*. DBtLj and DBtLjCg. (**B**) Clinical score. * p < 0.0001, D and DCg groups vs. DBtLj and DBtLjCg*.* (**C**) Histological score. *p* < 0.01, D and DCg groups *vs*. DBtLj and DBtLjCg. (**D**) Histological analysis of colon sections from DSS-induced colitis. Panels (a), (c) and (e) correspond to colon sections from mice receiving water (control), *C. glabrata* alone and BtLj, respectively. Panel (g) corresponds to colon sections from mice receiving DSS. Panel (i) corresponds to colon sections from mice receiving DSS + BtLj. Panel (K) represents colon sections from mice receiving *C. glabrata* + DSS. Panel (m) represents colon sections from mice receiving *C. glabrata* + BtLj + DSS. Colon sections from either D or DCg show tissue destruction (θ), important inflammatory cell infiltrates (δ) and oedema in the mucosa and submucosa of colon wall structures (φ). Scale bars represent 50 µm (a, c, e, g, i, k and m) and 10 µm (b, d, f, h, j, l and n).

In terms of inflammatory parameters, control mice (CTL), BtLj (*B. thetaiotaomicron* + *L. johnsonii*) and Cg (*C. glabrata*) groups, did not show any inflammatory signs. In the presence of DSS, the D (DSS) and DCg (DSS + *C. glabrata*) groups had a 15% loss in body weight starting from day 12 when compared to that of control mice (Fig. 4A). This decrease in body weight was inversely correlated with the increase in clinical score in the D and DCg groups (Fig. 4A,B). Although inflammatory parameters increased during the development of colitis and *C. glabrata* overgrowth, treatment with the two bacteria significantly decreased the clinical signs of inflammation, body weight and clinical score for inflammation in D or DCg mice.

Microscopic observation of colon sections from control groups (CTL, BtLj and Cg groups) showed an intact epithelium with no inflammatory cell infiltrates in the colon mucosa (Fig. 4C,D). In contrast, D or DCg mice showed histopathological changes consistent with an inflammatory process including epithelial damage, scattered inflammatory cell infiltrates in the mucosa and submucosa with the presence of oedema. However, the histopathological features were less severe in DBtLj and DCgBtLj mice than in D or DCg mice and some colon sections were entirely normal with no evidence of inflammatory changes suggesting that BtLj treatment protected mice from DSS-mediated colitis (Fig. 4C,D).

**Figure 5:**
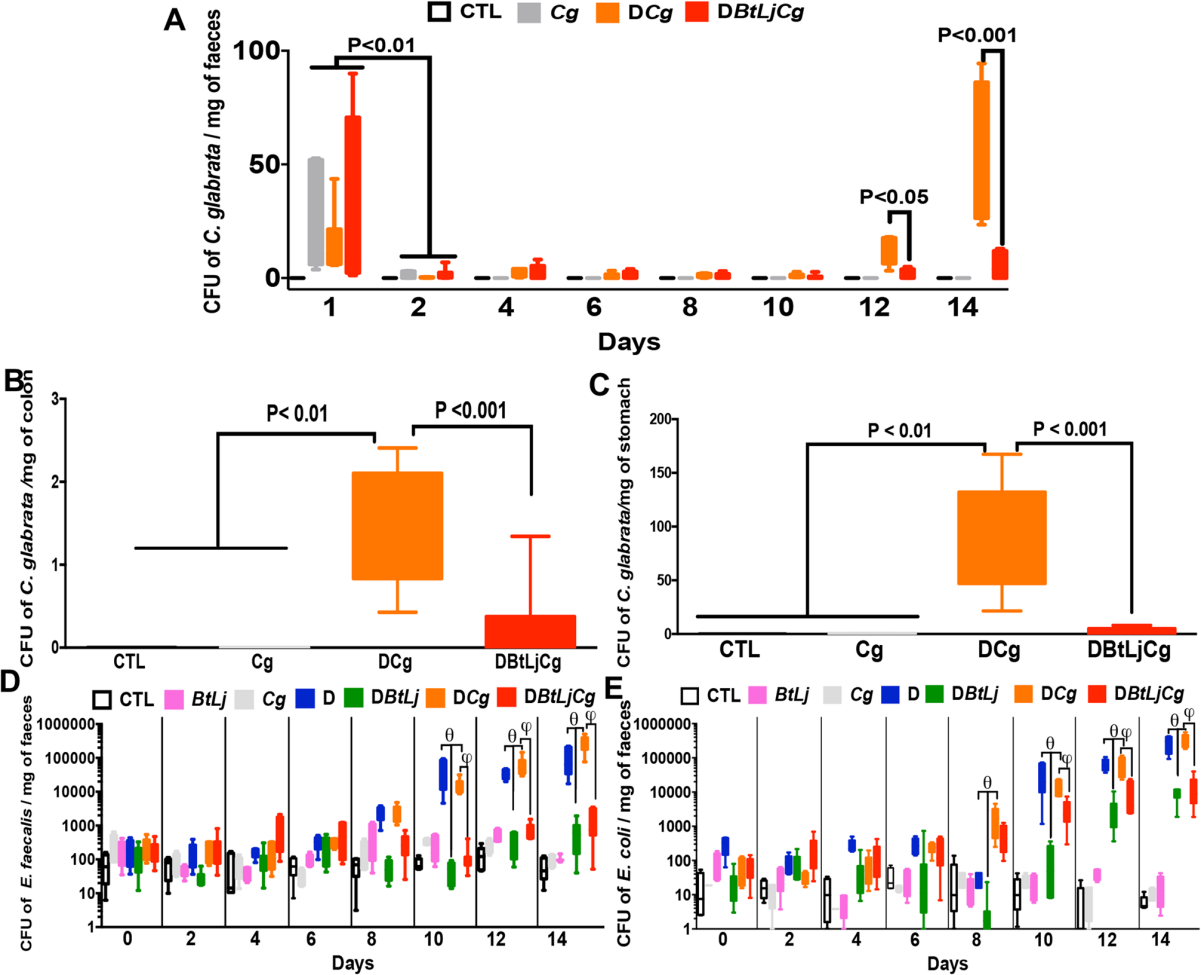
Effect of *B. thetaiotaomicron* and *L. johnsonii* on *C. glabrata* elimination from the gut and faecal aerobic bacteria. (**A**–**C**) Number of *C. glabrata* colonies recovered from the stools, stomach and colon. Data are the mean ± SD of eight mice per group from two independent experiments. (**D** and **E**) Determination of the number of viable *E. coli* and *E. faecalis* colonies recovered from stools. For all experiments, stool bacteria were collected from each tagged mouse on day 0 before DSS treatment and *C. glabrata* challenge. Data are the mean ± SD of eight mice per group from two independent experiments.

The number of *C. glabrata* colony-forming units (CFU) was counted in the stools and different segments of the digestive tract, in particular the colon and stomach. A significant decrease in *C. glabrata* CFU was observed in stool samples between day 1 and day 4 for all groups (Fig. 5A). This decrease continued until day 6 when significant elimination of *C. glabrata* was observed in the Cg group. Following DSS treatment, an increase in *C. glabrata* CFU was observed in the DCg group on days 12 and 14 and this fungal overgrowth was correlated with the development of colitis (Fig. 5A). In contrast, treatment with BtLj significantly decreased fungal overgrowth in mice. In addition, *C. glabrata* CFU were significantly reduced in the stomach and colon of DCgBtLj mice when compared to DCg mice (Fig. 5B,C). In terms of the impact of anaerobic bacteria on colitis, *E. coli* and *E. faecalis* populations increased significantly starting from day 8 to day 14 in D and DCg groups when compared to controls and this overgrowth occurred regardless of *C. glabrata* colonization. In contrast, a significant decrease in these two bacterial populations was observed in mice treated with BtLj (Fig. 5D,E).

### Effect of *B. thetaiotaomicron* and *L. johnsonii* on modulation of the innate immune receptor and cytokine expression and on induction of IgA in the colon

**Figure 6:**
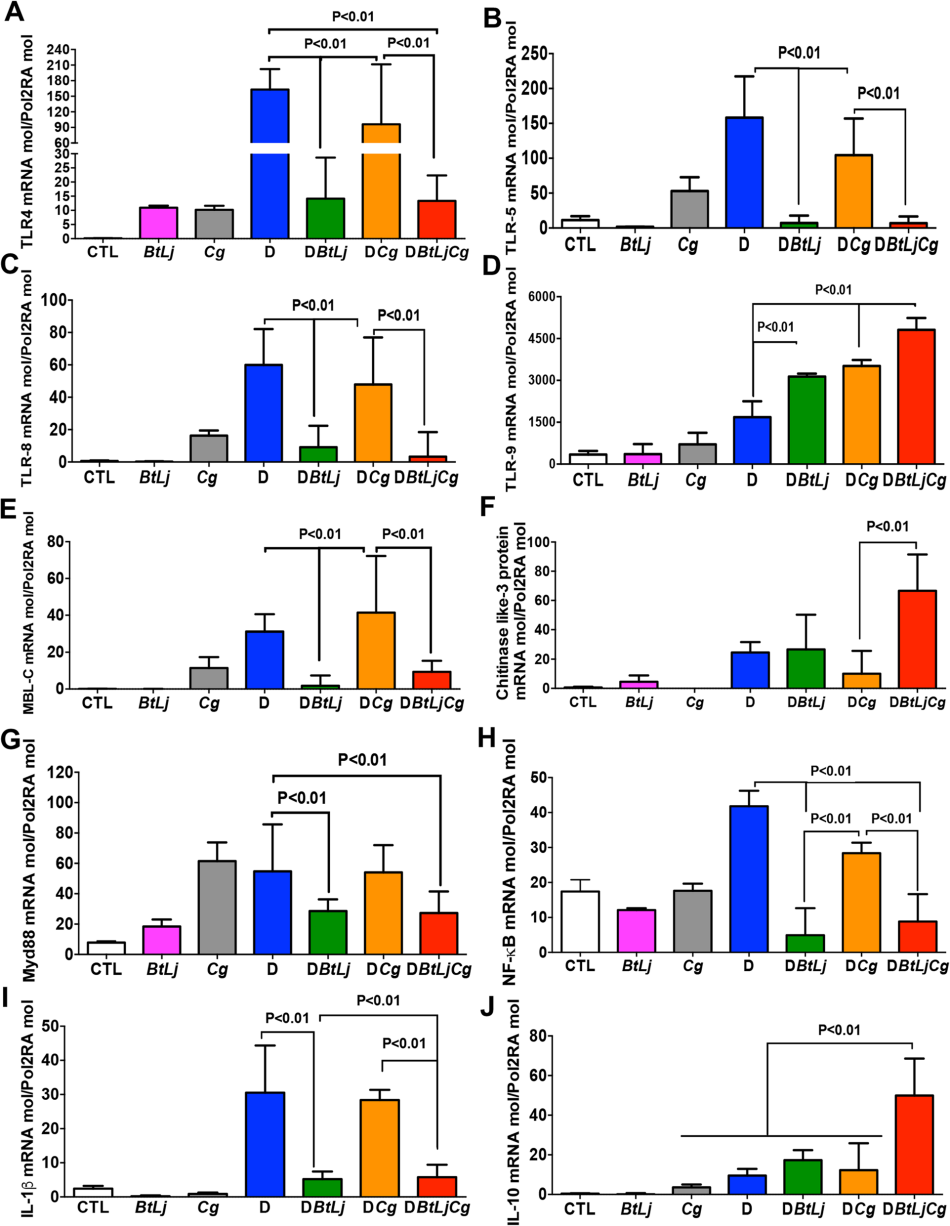
Receptor and cytokine expression in mice with colitis. (**A**–**J**) Relative expression levels of TLR4, TLR5, TLR8, TLR9, MBL-C, chitinase-like 3 protein, Myd88, NF-κB IL-1β and IL-10 mRNA in mouse colons. Data are the mean ± SD of eight mice per group from two independent experiments.

**Figure 7:**
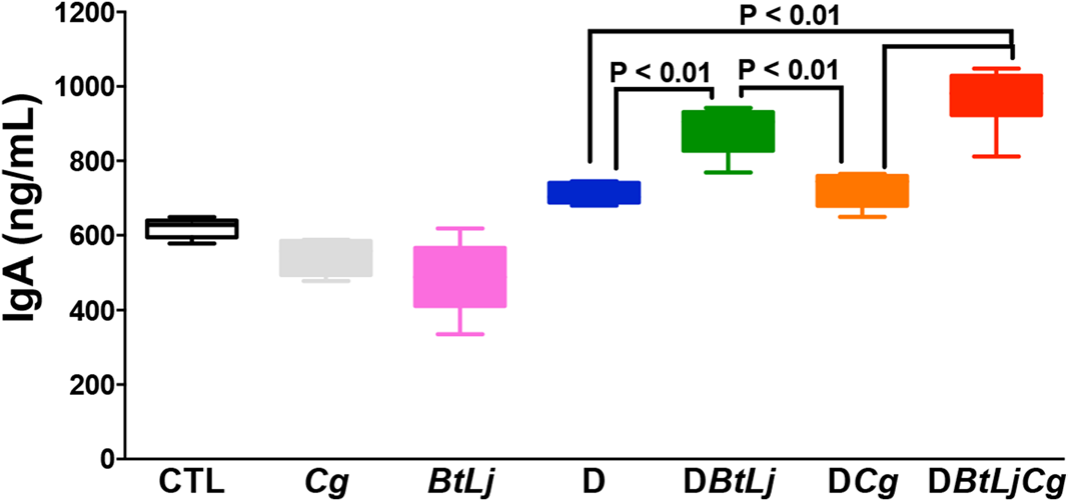
Secretory IgA in mouse colons. Data are the mean ± SD of eight mice per group from two independent experiments.

TLR and MBL-C play a crucial role in sensing *Candida* species and have a pivotal role in the pathogenesis of IBD. Following DSS treatment, significant increases in expression of TLR4, TLR5, TLR8, TLR9 and MBL-C were observed in D and DCg mice when compared to controls while BtLj treatment reduced TLR and MBL-C expression; the exception was the TLR9 transcript which was increased significantly in mice challenged with BtLj (Fig. 6). Expression of chitinase-like 3 protein, which is involved in the degradation and elimination of fungi, was highly increased in DCgBtLj mice. This high expression of chitinase-like 3 protein was correlated with the increased expression of TLR9 and elimination of *C. glabrata* from the gut. In terms of the signalling pathways mediated by TLR in particular, the MyD88-dependent pathway, which is common to all TLRs, and NF-κB expression increased significantly and an increase in expression of Myd88 and NF-kB signalling factors was observed following DSS treatment, while treatment with BtLj reduced this expression (Fig. 6). These data correlate with the expression of the pro-inflammatory cytokine IL-1β, which was decreased significantly in mice treated with BtLj, while the expression of the anti-inflammatory cytokine IL-10 was highly increased in these mice. In terms of IgA induction in the colon following BtLj treatment, a basal IgA concentration of approximately 600 ng/mL was observed in the control groups (CTL, Cg and BtLj). IgA level increased during the development of colitis, in particular in D or DCg mice where IgA levels were around 700 and 800 ng/mL, respectively (Fig. 7). However, the IgA level was even higher in groups treated with BtLj (800 and 1,000 ng/mL for DBtLj and DCgBtLj, respectively) (Fig. 7).

## Discussion

The intestinal microbiota act as a natural barrier against the proliferation of opportunistic pathogens including the opportunistic yeast *Candida*^1,2^. Bacteroidetes and Firmicutes are two dominant phyla, which mostly contain obligate anaerobic bacteria and are responsible for providing energy sources to colonic enterocytes and protection against pathogenic microorganisms^24^. Although the aetiology of IBD remains unclear, intestinal inflammation is often correlated with changes in the biodiversity of the gut microbiota in IBD patients^3^. These changes in the gut microbiota are referred to as dysbiosis^25^. Additionally, oxygen and reactive oxygen species play a crucial role in the pathogenesis of IBD by creating an imbalance between obligate and facultative anaerobes. Intestinal inflammation promotes an increase in release of haemoglobin carrying oxygen and reactive oxygen species into the intestinal lumen which creates a microenvironment for facultative anaerobes and a reduction in obligate anaerobes that leads to increased inflammation. The metabolic biofilm model shows that oxygen can play a crucial role in IBD dysbiosis by increasing the abundance of *E. coli* at the expense of the obligate anaerobe *B. thetaiotaomicron*^26^. In terms of pathogenic yeasts, it has been shown that intestinal inflammation promotes the overgrowth of *Candida* species, which in turn aggravates intestinal inflammation in mice^21,27^. Clinically, CD patients have high levels of ASCA and an abundance of *Candida* species in the gut when compared to healthy individuals. Additionally, in the DSS-induced colitis model, which mimics what happens in IBD patients, it has been reported that intestinal inflammation and *C. glabrata* overgrowth promote a modification of the gut microbiota biodiversity^14^. Among these aerobic bacteria, *E. coli* and *E. faecalis* populations are highly increased while two obligate anaerobic bacteria, *L. johnsonii* and *B. thetaiotaomicron* are reduced during the development of colitis, indicating that an alteration of the gut microbiota accelerates the disease process^15^. Furthermore, a decrease in these two anaerobic bacteria is correlated with the overgrowth of *C. glabrata* in the mouse gut. This observation led us to assess whether *B. thetaiotaomicron* and *L. johnsonii* can inhibit the growth of *Candida* species in vitro.

In the present study, we observed that co-incubation of *Candida* with *L. johnsonii* and *B. thetaiotaomicron* decreased the viability and growth of *Candida* species. These data are consistent with different studies showing that growth, adhesion and filamentation of *C. albicans* are inhibited by *Lactobacillus* species^28^. In addition, we showed that *B. thetaiotaomicron* induced the degradation of *C. glabrata* cell wall mannans. These data support those of a study showing that *B. thetaiotaomicron* utilizes complex machinery to digest and metabolize *C. albicans* cell wall mannans, enabling *B. thetaiotaomicron* to adapt to different competitive environments and to nutrients present in the intestinal tract^29^. Furthermore, we also observed that *L. johnsonii* produces enzymes with chitinase-like activity that promote the degradation of chitin and elimination of both *C. albicans* and *C. glabrata*. This in vitro approach reveals that these two anaerobic bacteria can inhibit the growth of *Candida* species through their antifungal enzyme activities.

In the DSS-induced colitis model, *L. johnsonii* and *B. thetaiotaomicron* were administered orally to mice for 5 days in order to restore the anaerobic bacteria and to promote fungal elimination. We observed a significant decrease in inflammatory parameters in terms of body weight, clinical score and histological score for inflammation in mice treated with anaerobic bacteria. In addition, a decrease in *E. coli* and *E. faecalis* and *C. glabrata* populations was observed in the mice indicating that these two anaerobic bacteria not only have a beneficial effect on *C. glabrata* elimination from the gut, but also on modulating intestinal inflammation and restoring the microbial balance.

IgA plays a crucial role in the elimination of pathogens by inhibiting their adherence and access to epithelial surfaces^30^. Germ-free mice have low levels of intestinal IgA whereas restoring their commensal microbiota promotes IgA production^31,32^. In our study, restoration of these two anaerobic bacteria was found to promote IgA secretion, which was correlated with a decrease in *E. coli*, *E. faecalis* and *C. glabrata* populations in mice.

Changes in gut microbiota biodiversity are known to affect the regulation of TLR and other intestinal receptors, in particular mannose-binding lectin (MBL)^10,33^. MBL is a collectin produced by hepatocytes and intestinal epithelial cells^33^. Sensing of the fungal cell wall by the MBL complex contributes to the elimination of *Candida* species from the gut^33,34^. Following DSS treatment and *C. glabrata* challenge, high expression of TLR4, 5, 8 and 9 and MBL-C was observed in the colon, indicating that the development of colitis and alterations in the gut microbiota drive TLR and MBL-C activation by different opportunistic bacteria and yeasts including *E. coli*, *E. faecalis* and *C. glabrata*. In contrast, treatment of mice with BtLj reduced the expression of TLR and MBL-C, except for TLR9 which was increased substantially. TLR9 is expressed intracellularly in immune cells and on the apical and basolateral membranes of intestinal epithelial cells^35^. Remarkably, this high TLR9 expression was correlated with low NF-κB transcripts in mice treated with BtLj. TLR9-deficient mice were highly susceptible to experimental colitis and had a lower NF-κB activation threshold when compared to wild-type and TLR2-deficient mice^36^.

IL-1β is a potent pro-inflammatory cytokine that is often upregulated in IBD patients and is involved in maintaining chronic gut inflammation, while the anti-inflammatory cytokine IL-10 has a crucial role in regulating inflammasome-mediated IL-1β production^37–39^. Patients with IL-10R deficiency develop severe IBD in the first months of life^40^. In the present study, the development of colitis and *C. glabrata* overgrowth were associated with high expression of IL-1β and down-regulation of IL-10. These findings were corroborated by high clinical and histological scores and an abundance of aerobic bacterial populations. In contrast, mice treated with BtLj displayed attenuated colitis, accompanied by a significant reduction in IL-1β and an increase in IL-10 transcripts. This IL-10 up-regulation was associated with increased chitinase-like 3 protein, which is involved in the degradation of fungal chitin. These data provide evidence that *L. johnsonii* and *B. thetaiotaomicron,* through their chitinase-like and mannosidase-like activities, can generate small mannose and chitin particles that are recognized by intestinal MBL-C and TLR and promote the activation of chitinase-like protein-1. This in turn enables chitin digestion and the generation of small sized chitin particles that drive IL-10 production via TLR-9 sensing, promoting the attenuation of colitis and *C. glabrata* elimination. These data are consistent with a previous study that demonstrated that the administration of fungal chitin activated chitinase-like protein-1, promoting *C. glabrata* elimination in a DSS-induced colitis model^14^.

In conclusion, *L. johnsonii* and *B. thetaiotaomicron* can interact directly with *Candida* species and induce degradation of the fungal cell wall, mediated via chitinase-like and mannosidase-like activities, which promotes inhibition of *Candida* species growth. In the DSS-induced colitis model, oral administration of *L. johnsonii* and *B. thetaiotaomicron* restored the imbalance between aerobic and anaerobic populations following DSS treatment and *C. glabrata* challenge and resulted in a significant reduction in inflammatory parameters revealed by a reduction in clinical and histological scores for inflammation. *L. johnsonii* and *B. thetaiotaomicron* also decreased pro-inflammatory mediators and enhanced anti-inflammatory cytokine responses. Modulation of cytokine expression is associated with high TLR9 expression and chitinase-like protein-1 activation, which promotes the elimination of *C. glabrata* from the gut. Overall, these findings provide evidence that *L. johnsonii* and *B. thetaiotaomicron* not only attenuated the development of colitis mediated by TLR9 but were also involved in the elimination of *C. glabrata* from the gut via chitinase-like and mannosidase-like activities.

## Methods

### Fungal and bacterial strains

*C. glabrata* BG2 wild-type (ATCC; Cg), *C. albicans* SC 5,314 (Ca) and bioluminescent *C. albicans* CEC749 Gluc strains were used in the present study^18,41^. Fungal culture was carried out in YPD medium (yeast extract 1%, peptone 1%, dextrose 1%) on a rotary shaker for 18 h at 37 °C. The culture obtained was then centrifuged at 2,500 rpm for 5 min and washed twice in PBS (phosphate-buffered saline). For bacterial culture, *B. thetaiotaomicron* and *L. johnsonii* were isolated from mouse stools on Bacteroides Bile Esculin agar and De Man, Rogosa and Sharpe (MRS) culture media, respectively, at 37 °C for 48 h under anaerobic conditions^14,15^. Identification of *B. thetaiotaomicron* and *L. johnsonii* was performed by MALDI-TOF mass spectrometry (Maldi-TOF, Microflex-Bruker Daltonics)^14^.

### Determination of the antifungal effect of *B. thetaiotaomicron* and *L. johnsonii* on *Candida* species

The viability of *C. albicans* cells was tracked in real-time using a bioluminescent yeast strain challenged with *B. thetaiotaomicron* and *L. johnsonii*. A suspension of 10^6^ bioluminescent *C. albicans* in 150 µL RPMI media was added to each well of a black 96-well plate (Greiner bio-one, Chimney well). *B. thetaiotaomicron* and/or *L. johnsonii* were then added to each well at a ratio of 1:1, 1:5 or 5:1 yeast-bacteria. Coelenterazine was then added to each well at a concentration of 2 μM. Bioluminescence kinetics were measured at 30 and 120 min and analysed with a FLUOstar Omega Fluorometer (BMG Labtech). The positive control consisted of *C. albicans* strain alone. The kinetics were also measured with a Xenogen device. Viability of *C. glabrata* in the presence of *B. thetaiotaomicron* and/or *L. johnsonii* was determined by serial dilution. A suspension of *C. glabrata* and *B. thetaiotaomicron* and/or *L. johnsonii* at 1:1, 1: 5 or 5:1 yeast-bacteria ratios was incubated for 30 or 120 min and an aliquot of 100 µL of each dilution was plated on Sabouraud dextrose agar and incubated for 24 h. For observation by confocal microscopy, *C. glabrata* cells were labelled with concanavalin A (ConA)-rhodamine and bacterial cells with calcein (Invitrogen, France). Specific slides (well 6.7 mm; Thermo Scientific, France) were used in this experiment and were examined by confocal microscopy (Zeiss LSM710, Zeiss Airyscan SR mode × 6,311.4).

### Antifungal activities of bacterial enzymes

To determine the chitinase activity of *L. johnsonii*, a suspension of 10^6^ yeasts in the presence of *L. johnsonii* (at a ratio of 1:1) was incubated overnight at 37 °C in 500 µL RPMI. After co-incubation, chitinase activity was measured as described previously^42^. Briefly, 0.5 mL of sample, 1 mL of acetate buffer (0.2 M at pH 5.0) and 5 mg of chitin-azur (Sigma, France) were mixed and incubated at 50 °C for 3 h. The samples were then centrifuged. The supernatant was read at 550 nm using a spectrophotometer. The positive control consisted of pure chitinase (chitinase from *Streptomyces griseus* at a concentration of 200 U/g; Sigma, France). For mannosidase activities of *B. thetaiotaomicron*, a suspension of 10^7^ yeasts in the presence of *B. thetaiotaomicron* (at a ratio of 1:1) was incubated overnight at 37 °C in 500 µL RPMI. The samples were then centrifuged and washed several times with PBS. Proteins and glycoconjugates were recovered by alkaline extraction under reducing conditions. The protein content of each extract was estimated using a bicinchoninic acid protein assay (Pierce) and was adjusted to the same protein concentration prior to analysis by SDS-PAGE on a 10% polyacrylamide gel^18^. The glycoproteins were then probed with either biotinylated lectin ConA (Sigma-Aldrich, France) or GNL (biotinylated galanthus nivalis; Vector Laboratories, California USA), both diluted 1:1,000. Horseradish peroxidase (HRP)-labelled streptavidin (1:2000 dilution) (Southern Biotech) was used to detect mannans. The glycoproteins of interest were revealed and detected using a reading device (Carestream, Image station, 4,000 MM Pro) and different exposures of the blots were performed (Supplementary data). For the determination of *C. glabrata* mannan levels by ELISA, a *C. glabrata* suspension of 10^5^ cells was co-incubated with 10^5^ *B. thetaiotaomicron* in 200 µL RPMI for 2 h or 48 h in 96-well plates (Greiner bio-one, Chimney well). After centrifugation, the supernatant was removed and the plate was saturated with 1% PBS-bovine serum albumin (BSA; Sigma-Aldrich, France) for 1 h. The supernatant was then removed and either ConA (Sigma-Aldrich, France) or GNL was added to each well at a concentration of 10 μg/mL for 1 h. After different washes with PBS, 100 μL of streptavidin-HRP was added to each well for 1 h. Absorbance was measured at 450 nm. Bacteria alone were used as controls.

### Animals

The animals used were 3–4-month-old wild-type female C57BL/6 mice, certified disease-free (Janvier Laboratories, France). The mice were housed in the pet store of the Faculty of Medicine, Lille. The temperature of the room was maintained at 21 °C and the mice had free access to water and food with exposure to light 12 h/day. All animal experiments were approved by the subcommittee for Research Animal Care, Regional Hospital Centre, Lille, France (00550.05) and in accordance with institutional (86/609/CEE) and European guidelines for the care and use of laboratory animals. The animals were tagged and separated into seven groups. Control groups were: CTL group, receiving only water; Cg group, receiving only *C. glabrata*; and BtLj group, receiving only *B. thetaiotaomicron* + *L. johnsonii*. The experimental groups were: D (group treated with DSS only), which represented the control group for intestinal inflammation; DCg (DSS + *C. glabrata*), which served as a control group for fungal colonization and intestinal inflammation; DBtLj (DSS + *B. thetaiotaomicron* + *L. johnsonii*), group to assess the effect of anaerobic bacteria on intestinal inflammation; and DCgBtLj (DSS + *C. glabrata* + *B. thetaiotaomicron* + *L. johnsonii*), group of interest.

### Inoculum preparation and induction of colitis

Mice were given 200 µL PBS containing 10^8^ live *C. glabrata* cells on day 1 by oral gavage. From day 1 to day 14, mice were also treated with DSS (36–50 kDa; stock solution 2%, diluted solution 1.5%, MP Biomedicals, LLC, Germany) in drinking water in order to induce colitis. For restoration of the two anaerobic bacterial populations, mice were given 10^6^ live *B. thetaiotaomicron* and/or 10^6^ live *L. johnsonii* in 200 µL PBS orally and daily for 5 days, starting on day 1. The presence of *C. glabrata* in the gut was assessed by counting the number of colonies in faeces (approximately 0.1 g/sample) collected from each tagged animal on day 14. Faecal samples were suspended in 1 mL PBS and then cultured on Candi-Select medium (Bio-Rad Laboratories, France). The results are shown as colony-forming units (CFU)/mg faeces^21^. For the isolation of bacteria, mouse faecal samples were collected and serial dilutions were performed daily. The samples were cultured on non-selective bacterial media (AC agar) focusing on the most representative cultivable anaerobic and aerobic bacteria that can undergo changes during intestinal inflammation. Isolation of aerobic and anaerobic bacteria was carried out on MacConkey (MCK) and Bile Esculin azide culture media, respectively. Identification of bacteria isolated on selective media was performed by MALDI-TOF mass spectrometry (Maldi-TOF; Microflex-Bruker Daltonics)^14^.

### Clinical and histological scores for inflammation

Body weight of each tagged mouse was measured daily and the presence of blood in the rectum and stool consistency were also determined^14^. Clinical scores, as described previously, were analysed independently by two investigators blinded to the protocol^27,41^. Two scores (stool consistency and bleeding) were added, resulting in a total clinical score ranging from 0 (healthy) to 12 (maximum colitis activity). For the histological score, the entire colon from the caecum to the anus was collected. The colon samples were fixed overnight in 4% paraformaldehyde-acid, embedded in paraffin, cut in a microtome (Leica RM2245, Leica Biosystems, Germany) and affixed onto slides for histological staining. The colon sections (4 µm thick) were stained with periodic acid–Schiff stain (Merck, France) and then viewed with a digital slide scanner (Axio-Scan.Z1, Zeiss, Jena, Germany). Histological scoring was performed by two independent investigators blinded to the protocol. The score was based on the infiltration of inflammatory cells into the submucosa and epithelial damage of the colon and ranged from 0 (no changes) to 6 (extensive cell infiltration and tissue damage)^14,43^.

### Secretory IgA in the mouse colons

Mouse colons were flushed with 2 mL PBS and the intestinal content was vortexed and assayed for IgA using an ELISA kit (Invitrogen, France)^30^. Briefly, the first antibody (purified monoclonal anti-mouse IgA) was fixed to the bottom of the wells of the 96-well plate (overnight at 4 °C). After different washings, mouse colon contents were deposited in each well for 2 h and the wells were then washed. The second antibody (HRP-conjugated monoclonal anti-mouse IgA) was added to each well for 1 h. After washing, substrate solution was added and incubated for 15 min followed by the addition of stop solution. Optical density was measured with a spectrophotometer at 450 nm (Biorad, France). The results were analysed by normalizing to standard concentrations of IgA.

### Real-time mRNA quantification of innate immune receptors

Extraction of total RNA from the colon was carried out using a commercial kit (Nucleospin RNA/Protein; Macherey–Nagel, France). RNA was quantified by spectrophotometry (Nanodrop; Nyxor Biotech, France). mRNA reverse transcription was performed in a final volume of 20 µL from 1 µg total RNA (high capacity cDNA RT kit; Applied Biosystems). Fast SYBR green (Applied Biosystems) was used in PCR to amplify the cDNA in a one-step system (Applied Biosystems). SYBR green dye intensity was assessed using one-step software. All results were normalized to the reference gene *POLR2A*^15,33^.

### Statistical analysis

All data are presented as the mean ± standard deviation (SD) of individual experimental groups. Pairs of groups were compared using the Mann–Whitney U test. Differences were considered significant when the P value was as follows: *p* < 0.05; *p* < 0.01; *p* < 0.001. Statistical analyses were carried out using Prism 4.0 from GraphPad and XLSTAT.

## Supplementary information

Supplementary Information.
