# Supplementary material for: Bacteroides thetaiotaomicron and Lactobacillus johnsonii modulate intestinal inflammation and eliminate fungi via enzymatic hydrolysis of the fungal cell wall
Source: Sci Rep. 2020 Jul 13;10:11510. doi: 10.1038/s41598-020-68214-9 (PMC7359362; doi:10.1038/s41598-020-68214-9)
Supplement: Supplementary file 1 — Supplementary Information. [file 41598_2020_68214_MOESM1_ESM.docx]

***Bacteroides thetaiotaomicron* and *Lactobacillus johnsonii* modulate intestinal inflammation and eliminate fungi via enzymatic hydrolysis of the fungal cell wall**

Rogatien Charlet, Clovis Bortolus, Boualem Sendid, Samir Jawhara

**Methods**

***C. glabrata* strain and culture conditions**

The strain used in the present study was *C. glabrata* wild-type (ATCC; Cg WT). Fungal culture was carried out in Sabouraud liquid medium on a rotary shaker for 48 h at 37° C. The yeast culture was then centrifuged at 3500 rpm for 5 min and washed twice in PBS (phosphate-buffered saline).

**Extraction of cell wall mannan from *C. glabrata***

Extraction of cell wall mannan from *C. glabrata* was carried out according to the method described by Faille et al.^1^ Briefly, after several washes of *C. glabrata* cells with PBS, 2 mL of 0.02 M citrate buffer, pH 7.0, was added to the *C. glabrata* pellet. This suspension was then autoclaved and centrifuged at 4000 rpm for 15 min. The supernatant was transferred to a new falcon tube and an equivalent volume of Fehling's solution was added (copper sulphate solution and sodium-potassium tartrate). After centrifugation at 3000 rpm for 15 min, the pellet was collected in 10 mL of 3 M HCL. A large volume of methanol /acetic acid (at a ratio 8:1) was added to the pellet. The precipitate obtained was washed several times in this same solution. The pellet was recovered, washed in methanol and then dried. The dried pellet was then diluted in 2 mL distilled water. The concentration of mannan was determined according to the method of Faille et al. using 5% phenol (Sigma, France) and sulphuric acid (Sigma, France).^1^ For determination of mannan degradation, 1 mL containing 100 µg mannan derived from *C. glabrata* was added to 10^7^ *B. thetaiotaomicron* cells. After 2 or 48 h incubation, the samples were then centrifuged and the supernatants collected. The mannoprotein content of each sample was estimated using a bicinchoninic acid protein assay (Pierce) and adjusted to the same protein concentration prior to analysis by SDS-PAGE on a 10% polyacrylamide gel.^2^ The mannoproteins were probed with either biotinylated lectin ConA (Sigma-Aldrich, France) or GNL (biotinylated galanthus nivalis; Vector Laboratories, California USA), both diluted 1:1000. Horseradish peroxidase (HRP)-labelled streptavidin (1:2000 dilution) (Southern Biotech) was used to detect mannans.

**Results**

**Degradation of cell wall mannan extracted from *C. glabrata* by *B. thetaiotaomicron***

Cell wall mannan was extracted from *C. glabrata* and incubated with *B. thetaiotaomicron.* Degradation of mannan by *B. thetaiotaomicron* was determined by Western blot. In these experiments, mannan extracted from *C. glabrata* alone forms a smear in SDS-acrylamide gels as do all other heavily glycosylated yeast glycoproteins.^3^ The mannan smear was no longer visible after incubation of mannan with *B. thetaiotaomicron* for 48 h. These data correlate with those showing degradation of cell wall mannan after co-incubation of *C. glabrata* with *B. thetaiotaomicron* and show that *B. thetaiotaomicron* is able to induce degradation of *C. glabrata* cell wall mannan (Fig. 1).


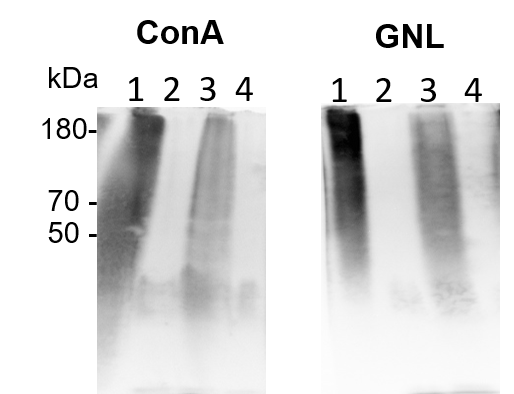


**Figure 1: Analysis by Western blot. Degradation of cell wall mannan extracted from *C. glabrata* by *B. thetaiotaomicron*. Mannan extracted from *C. glabrata* was labelled with either ConA or GNL.**  Line 1: mannans extracted from *C. glabrata*. Line 2: extract of *B. thetaiotaomicron*. Line 3: mannans from *C. glabrata* incubated with *B. thetaiotaomicron* for 2 h. Line 4: mannans from *C. glabrata* incubated with *B. thetaiotaomicron* for 48 h.

**Expression of IL-6 in the colons of mice treated with *L. johnsonii* and *B. thetaiotaomicron***

IL-6 expression was determined in colon samples from the different murine groups. IL-6 expression increased in DSS and DSS+*C. glabrata* groups when compared to that in the control groups. In contrast to IL-1β, treatment of mice with *L. johnsonii* and *B. thetaiotaomicron* did not decrease IL-6 expression. The increase in IL-6 in the colons of mice treated with *L. johnsonii* and *B. thetaiotaomicron* was not statically significant when compared to that in DSS and DSS+*C. glabrata* mice (Fig. 2).


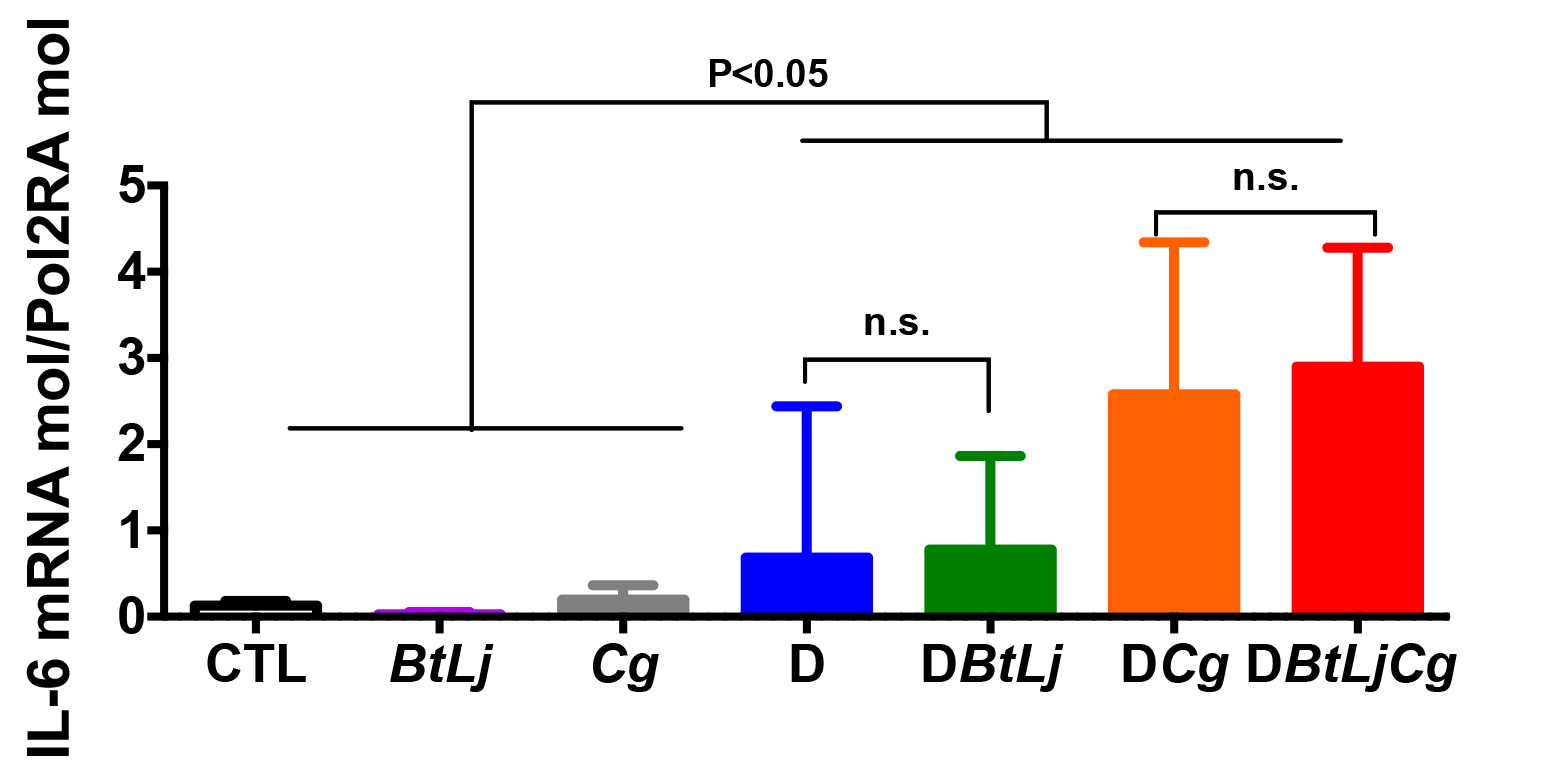


**Figure 2: Relative expression level of IL-6 in mouse colons.** No significant difference (n.s.) between group D and DBtLj. No significant difference (n.s.) between the group DCg and DBtLjCg. Data are the mean ± SD of eight mice per group from two independent experiments.

**References**

1. Faille, C. *et al.* Immunoreactivity of neoglycolipids constructed from oligomannosidic residues of the Candida albicans cell wall. *Infect Immun* **58**, 3537-3544 (1990).

2. Jawhara, S. *et al.* Murine model of dextran sulfate sodium-induced colitis reveals Candida glabrata virulence and contribution of beta-mannosyltransferases. *J Biol Chem* **287**, 11313-11324, doi:10.1074/jbc.M111.329300 (2012).

3. Conde, R., Cueva, R., Pablo, G., Polaina, J. & Larriba, G. A search for hyperglycosylation signals in yeast glycoproteins. *J Biol Chem* **279**, 43789-43798, doi:10.1074/jbc.M406678200 (2004).
